# Mapping child maltreatment risk: a 12-year spatio-temporal analysis of neighborhood influences

**DOI:** 10.1186/s12942-017-0111-y

**Published:** 2017-10-18

**Authors:** Enrique Gracia, Antonio López-Quílez, Miriam Marco, Marisol Lila

**Affiliations:** 1 Department of Social Psychology, University of Valencia, Av. Blasco Ibáñez, 21, 46010 Valencia, Spain; 2 Department of Statistics and Operations Research, University of Valencia, C/Doctor Moliner, 50, 46100 Burjassot, Valencia Spain

**Keywords:** Child maltreatment, Neighborhood influences, Bayesian spatio-temporal modeling, Disease mapping, Spatial inequality, Small-area study, Area-specific risk estimation

## Abstract

**Background:**

‘Place’ matters in understanding prevalence variations and inequalities in child maltreatment risk. However, most studies examining ecological variations in child maltreatment risk fail to take into account the implications of the spatial and temporal dimensions of neighborhoods. In this study, we conduct a high-resolution small-area study to analyze the influence of neighborhood characteristics on the spatio-temporal epidemiology of child maltreatment risk.

**Methods:**

We conducted a 12-year (2004–2015) small-area Bayesian spatio-temporal epidemiological study with all families with child maltreatment protection measures in the city of Valencia, Spain. As neighborhood units, we used 552 census block groups. Cases were geocoded using the family address. Neighborhood-level characteristics analyzed included three indicators of neighborhood disadvantage—neighborhood economic status, neighborhood education level, and levels of policing activity—, immigrant concentration, and residential instability. Bayesian spatio-temporal modelling and disease mapping methods were used to provide area-specific risk estimations.

**Results:**

Results from a spatio-temporal autoregressive model showed that neighborhoods with low levels of economic and educational status, with high levels of policing activity, and high immigrant concentration had higher levels of substantiated child maltreatment risk. Disease mapping methods were used to analyze areas of excess risk. Results showed chronic spatial patterns of high child maltreatment risk during the years analyzed, as well as stability over time in areas of low risk. Areas with increased or decreased child maltreatment risk over the years were also observed.

**Conclusions:**

A spatio-temporal epidemiological approach to study the geographical patterns, trends over time, and the contextual determinants of child maltreatment risk can provide a useful method to inform policy and action. This method can offer a more accurate description of the problem, and help to inform more localized prevention and intervention strategies. This new approach can also contribute to an improved epidemiological surveillance system to detect ecological variations in risk, and to assess the effectiveness of the initiatives to reduce this risk.

## Background

 Child maltreatment is a major social, public health, and human rights problem, with severe, far-reaching, and long-lasting consequences. Its impact on victims’ physical, mental, and reproductive health, behavioral problems, or education attainment; its role in the intergenerational transmission of violence; and its elevated costs for the criminal, health, and social welfare systems, poses a high burden on society [1–4]. Child maltreatment is a global phenomenon, and its prevalence in high-income countries remains high and is considered a leading cause of health inequality and social injustice [4–7]. From a public health perspective, child maltreatment is, however, a preventable problem as potentially risk-modifying factors can be identified and targeted in preventive interventions.

Child maltreatment research has typically focused on individual or family risk factors, but ‘place’ also matters in understanding prevalence variations and inequalities in child maltreatment risk [8–12]. A growing body of research is increasingly recognizing the importance of the context in which families live, linking neighborhood characteristics and processes—such as poverty, disorder and crime, immigrant concentration, social impoverishment, or diminished social control—to child maltreatment [13–32]. However, studying neighborhood influences on child maltreatment presents important challenges, and there are still some shortcomings in the available literature, which we address in the present study.

Child maltreatment and neighborhood risk factors are not equally distributed spatially, and it is to some extent surprising that the use of spatial analysis techniques and disease mapping methods to analyze geographical patterns of child maltreatment risk, and whether these patterns are associated with neighborhood-level explanatory variables, has been almost non-existing. In this regard, and despite the substantial body of research showing an association between neighborhood characteristics and child maltreatment, most studies examining neighborhood influences fail to take into account the implications of the spatial dynamics of neighborhoods. For example, important issues such as the similarity and influence between neighboring areas, are not appropriately addressed in most studies analyzing neighborhood influences on child maltreatment [10, 11, 25]. Neighborhood risk factors that have been associated with child maltreatment are usually clustered in space, and therefore a spatial analytical approach is particularly appropriate for the study of their influences on the spatial variations of child maltreatment. Furthermore, when analyzing neighborhood influences on ecological variations in child maltreatment risk, the temporal dimension must also be taken into account. Neighborhoods characteristics may change over time and, therefore, their influence on child maltreatment risk can also change over time [10]. In this regard, adding the temporal dimension is a key in identifying and tracking trends in child maltreatment risk—for example, stable high or low risk areas, or areas of increasing or decreasing risk over time. Finally, most studies exploring neighborhood influences on child maltreatment do not provide small-area-specific risk estimations, which limits their relevance to inform more localized intervention and prevention strategies. A new generation of ecological studies needs to take into account the spatial and temporal dimensions to better understand small-area variations in child maltreatment risk.

Bayesian spatio-temporal modeling provides an adequate methodological framework to overcome the above limitations in studying neighborhood influences on child maltreatment risk variations. This analytical approach, allows to incorporate geographical and temporal information to provide more reliable area-specific risk estimates than other non-Bayesian methods, by addressing important methodological issues such as modeling small area counts, spatial auto-correlation, or overdispersion, that can bias estimates if ignored [33–39]. Although Bayesian spatio-temporal disease mapping is common in other public health and epidemiological areas, this approach is relatively new in the area of family violence [40–44]. So far, only a handful of studies conducted in the US have addressed the spatial and temporal dimensions to study neighborhood influences on child maltreatment with appropriate methodologies [40, 42, 44]. However, these studies are usually low-resolution ones, using larger geographical areas, such as counties, zip codes, or census track, which somehow limits their potential to inform more localized interventions. On the other hand, high-resolution studies using small-area geographical units offer a finer neighborhood characterization, and provide specific risk estimations for small areas, which increases their potential to inform highly localized policies targeting high-risk areas [36, 38, 39].

Based on a social disorganization theoretical framework [43, 45–48] in this study we analyze the influence of a set of neighborhood characteristics on local patterns of substantiated child maltreatment over a 12-year period at the small-area level. Neighborhood-level characteristics analyzed include three indicators of neighborhood concentrated disadvantage—neighborhood economic status, neighborhood education level, and levels of policing activity—, immigrant concentration, and residential instability. As far as we are aware, the present study is the first to conduct a high-resolution small-area study on the spatio-temporal epidemiology of child maltreatment risk using Bayesian spatio-temporal modeling and disease mapping methods. Our study is conducted in a European city which also provides a ground for comparison to US cities where most of the research on neighborhood influences on child maltreatment has been conducted.

## Methods

### Variables

The study was conducted in the city of Valencia, the third largest city of Spain with a population of 736,580. As neighborhood proxies, we used the 552 census block groups into which the city is divided. This was the minimum administrative unit available, with an average of 1334 residents, and ranging from 630 to 2845. To capture temporal trends, data for 12 years were used, from 2004 to 2015. Data for cases of substantiated child maltreatment were provided by Valencia Child Protection Services. Covariates for each census block group were provided by the Valencia Statistics Office and the Valencia Police Department.

#### Outcome variable

Number of families with child maltreatment protection measures. Data of all families with child protection measures from 2004 to 2015 were collected—no computerized and systematized data were available before 2004. The data we used correspond to all official cases of substantiated child maltreatment in the city of Valencia during the period of the study, to which a child protection measure is associated after the maltreatment and the risk for the child is established. Child maltreatment refer to any type of child maltreatment, including physical, psychological, or sexual abuse, as well as neglect. Perpetrators were parents or legal tutors–child abuse by other parties, such as non-related adults, are dealt by other agencies and are not considered in this study. The data provided by the Child Protection Services did not allow to distinguish between child maltreatment types or perpetrators. Protection measures are issued by the Child Protection Services for all substantiated cases of child maltreatment, and may include a range of measures such as home visiting, family support programs, family or residential foster care, or adoption. Data for this study were 1799 families with child maltreatment protection measures. To avoid data dependency, cases were ‘unique’ families, meaning that a family was only included the first time they received a child maltreatment protection measure. Data were geocoded using the family address, and we counted the cases in each census block group for each of the 12 years in the study period.

#### Covariates

*Neighborhood concentrated disadvantage. Economic status*: Neighborhood economic status was measured using the average cadastral property value. This value is set by the City Hall and is used to establish local taxes. *Education level*: The average education level of neighborhood residents in each census block group was measured on a 4-point scale, where 1 = less than primary education, 2 = primary education, 3 = secondary education, 4 = college education. *Policing activity*: An indirect measure of public disorder and crime was measured through police officers’ assessments of their policing activity. Senior police officers provided an index of policing activity that included interventions in drug-related crime, public disorder such as drunkenness and fights, vandalism, homeless people and truancy. The index was structured as a 5-item scale, and each item ranged from 0—very low level of interventions—to 4—very high level of interventions. This measure of policing activity has been associated in previous studies with other types of family violence such as intimate partner violence [43], as well as with a number of neighborhood-level characteristics, such as neighborhood disorder, low socioeconomic status, and high immigrant concentration [49, 50]. Cronbach’s alpha was .74.

*Immigrant concentration*: Using census data, immigrant concentration was measured as the percentage of immigrant population in each census block group.

*Residential instability*: An index of residential mobility, based on census data, was used as the proportion—rate per 1000 inhabitants—of the population who had moved into or out of each census block group during the previous year—for example, residential instability for 2015 captures all population movements occurred in 2014.

### Statistical analysis

The outcome variable was the number of families with child maltreatment protection measures, corresponding to all substantiated cases of child maltreatment in the city during the period of the study. We use, therefore, a conditionally independent Poisson distribution based on the count of families in each census block group in the 12 years of the study:

$$y_{it} |\eta_{it} \sim\;Po\left( {E_{it} \exp (\eta_{it} )} \right), \quad i = 1, \ldots ,552 \quad t = 1, \ldots ,12$$

where $E_{it}$ is a fixed quantity that accounts for the expected number of families with child maltreatment protection measures, in proportion to the total number of families, in census block group $i$ in year *t*; $\eta_{it}$ is the log-relative risk for every area and year.

We used different models for $\eta_{it}$ with an increasing level of complexity, from a Poisson regression model to a spatio-temporal autoregressive model. First, model 1 only included all covariates—that is, economic status, education level, policing activity, residential instability, and immigrant concentration.

$$\eta_{it} = \mu + X_{it} \beta$$

where $\mu$ is the intercept, $X_{it}$ is the vector of covariates, and $\beta$ is a vector of regression coefficients.

Model 2 was specified as a spatial model; it included all covariates and added unstructured and structured random effects. The unstructured random effect accounted for spatial heterogeneity or overdispersion, while the structured random effect accounted for the spatial effect:

$$\eta_{it} = \mu + X_{it} \beta + \phi_{i} + \theta_{i}$$

where $\phi_{i}$ represents the spatially structured term, and $\theta_{i}$ the spatially unstructured term.

Model 3 included the previous terms and incorporated an unstructured temporal effect:

$$\eta_{it} = \mu + X_{it} \beta + \phi_{i} + \theta_{i} + \alpha_{t}$$

where $\alpha_{t}$ accounts for the temporal heterogeneity. This model, however, does not account for past cases of child maltreatment—that is, temporal dependency.

Finally, model 4 included a spatio-temporal effect. To this end, we followed an autoregressive approach [51], combining autoregressive time series and spatial modeling. We defined a spatio-temporal structure in which the relative risks are both spatially and temporally dependent.

$$\eta_{i1} = \mu + X_{it} \beta + \alpha_{1} + \left( {1 - \rho^{2} } \right)^{ - 1/2} \cdot\left( {\phi_{i1} + \theta_{i1} } \right)$$

$$\eta_{it} = \mu + X_{it} \beta + \alpha_{t} + \rho \cdot\left( {\eta_{{i\left( {t - 1} \right)}} - \mu - \alpha_{t - 1} } \right) + \phi_{it} + \theta_{it}$$

The first equation defines the log-relative risk for the first year observed (2004) and the second equation defines the log-relative risk for the following years. In both, $\alpha_{t}$ is the mean deviation of the risk in the year *t*, $\rho$ represents the temporal correlation between years, and $\phi_{it}$ and $\theta_{it}$ refer to structured and unstructured spatial random effects, respectively.

Models were specified following a Bayesian approach. Therefore, we assigned appropriate prior distributions for all parameters. We assigned vague Gaussian distributions for the fixed effects $\beta$; $\mu$ was specified as an improper uniform distribution; unstructured effects were modeled as a normal distribution $N\left( {0,\sigma^{2} } \right)$ in the different models ($\theta$ and $\alpha$). Structured effects ($\phi$) were specified by a conditional spatial autoregressive (CAR) model [52] defined as follows:

$$\phi_{i} |\phi_{ - i} \sim\;N\left( {\frac{1}{{n_{i} }}\mathop \sum \limits_{j\sim\;i} \phi_{j} ,\frac{{\sigma_{\phi }^{2} }}{{n_{i} }}} \right)$$

where $n_{i}$ is the number of neighboring areas of each census block group *i*, $\phi_{ - i}$ represents the values of the $\phi$ vector except the component *i*, $\sigma_{\phi }$ is the standard deviation parameter, and $j\sim\;i$ indicates all units *j* that are neighboring areas of census block group *i.* Finally, and following the structure of the hierarchical Bayesian models, hyperparameters $\sigma$ were specified by uniform distributions $U\left( {0,1} \right)$ in the models.

Bayesian estimations were performed using Markov Chain Monte Carlo simulation techniques with the software R and the WinBUGS package. 100,000 iterations were generated, discarding the first 10,000 as a burn-in period. Models were compared by the Deviance Information Criterion (DIC) [53]. This measure of fit assumes that models with smaller DIC should be preferred to models with larger DIC. Following this criterion, the model with smaller DIC was chosen. Differences in DIC between 5 and 10 are considered substantial; whereas differences of more than 10 units clearly indicate that the model with the higher DIC should be ruled out.

To ensure robustness of the results, we checked convergence with the convergence diagnosis $\hat{R}$ [54] which was near to 1.0 for all parameters. A sensitivity analysis was also performed on prior distributions of hyperparameters, with consistent results.

## Results

Table 1 summarizes the descriptive statistics of the variables in the study. The neighborhood-level economic status, based on the average cadastral property value, had a mean of 26,320 € [standard deviation (SD) = 13,046], with wide variation across neighborhoods, ranging from 7943€ to 98,560€. The average neighborhood education level, corresponded to secondary education (Mean = 3; SD = .33). Policing activity had a mean of 7.16 (SD = 3.99), again with wide variations across neighborhoods ranging from 0 to 19. The mean of neighborhood residential instability was 200 (SD = 65.96) meaning that, in average, 200 people moved into or out of a census block group in a specific year. Neighborhood immigrant concentration had a mean of 13.3%, ranging from just 1 to 51%, which means that in some neighborhoods over half of the population were immigrant. Finally, the outcome variable, families with child protection measures, ranged from 0 cases of substantiated child maltreatment in some neighborhoods to a maximum of 7 families per census block group in a single year with child protection measures after child maltreatment was substantiated.

**Table 1:** Variables (mean, standard deviation, minimum and maximum values) at the census block group and year level

Variable	Mean (SD)	Min	Max
Economic status (€)	26,320 (13,046)	7943	98,560
Education level	3.155 (.33)	2.39	3.86
Policing activity	7.16 (3.99)	0	19
Residential instability	200 (65.96)	4.2	771.3
Immigrant concentration (%)	13.28 (6.92)	1.03	51.47
Child protection records	0.26 (.57)	0	7

After conducting the four Bayesian Poisson regression models, we analyzed the DIC values (Table 2). Model 1, which included only the covariates, showed the worst fit (DIC = 8517.9). Once we introduced both structured and unstructured spatial random effects in model 2, the DIC decreased significantly (DIC = 8164.9). In model 3, which included an unstructured temporal effect, the DIC slightly increased to 8166.8. Finally, model 4, an autoregressive model, despite being the most complex had the lowest DIC value (8126.1), 38 units lower than model 2, and was therefore chosen as the final model. The sign of the covariate estimations (positive or negative) remains invariant in the different models, ensuring the stability of the effects.

**Table 2:** Results of different spatial and spatio-temporal regression Bayesian models for child maltreatment risk. Posterior mean, standard deviation (SD) and the 95% credible interval (CI) of all parameters

	Model 1 (β)			Model 2 (β + spatial heterogeneity + spatial effect)			Model 3 (β + spatial heterogeneity + spatial effect + temporal heterogeneity)			Model 4 (spatio-temporal autoregressive model)		
	Mean	SD	95% CI	Mean	SD	95% CI	Mean	SD	95% CI	Mean	SD	95% CI
Intercept	4.055	.289	3.500, 4.615	4.335	.516	3.320, 5.328	4.284	.520	3.916, 5.304	4.135	.500	3.274, 5.127
Economic status^a^	−.021	.003	−.027, −.015	−.016	.004	−.024, −.009	−.016	.004	−.023, −.009	−.016	.004	−.023, −.008
Education level	−1.261	.091	−1.431, −1.083	−1.464	.161	−1.745, −1.123	−1.418	.164	−1.746, −1.096	−1.391	.157	−1.690, −1.122
Policing activity	.026	.006	.014, .038	.036	.011	.012, .053	.035	.012	.012, .057	.031	.011	.009, .053
Residential instability	.000	.001	−.001, .001	.000	.001	−.001, .001	.000	.001	−.001, .001	.000	.001	−.001, .001
Immigrant concentration	.003	.005	−.006, .013	.005	.006	−.005, .016	.005	.005	−.005, .016	.009	.006	−.003, .020
σ_θ_				.329	.084	.136, .468	.320	.095	.063, .456	.234	.045	.162, .333
σ_ϕ_				.781	.115	.541, .979	.787	.118	.552, .976	.257	.062	.149, .391
σ_α_							.023	.019	.001, .070	.021	.019	.001, .070
ρ										.903	.031	.827, .946
DIC	8517.9			8164.9			8166.8			8126.1		

*SD* standard deviation, *CrI* credible interval, *DIC* deviance information criterion

σ_θ_ standard deviation unstructured term

σ_ϕ_ standard deviation spatially structured term

σ_α_ Standard deviation temporally unstructured term

^a^This variable was included as the cadastral value divided by 1000 to solve computational problems with the prior distributions assigned to fixed effects

For model relevance, we consider posterior probability distributions of the regression coefficients (β) of being different from zero. The probability of being positive was higher than 90% for policing activity and immigrant concentration. The probability of being negative was higher than 90% for economic status and education level. These results indicate that the risk of substantiated child maltreatment was particularly high in neighborhoods with low economic status and education level, and with high levels of policing activity and immigrant concentration. Residential instability, however, did not show a relevant association with substantiated child maltreatment.

To know the relative influence of the four neighborhood variables that were relevant for the final model, our results can be interpreted in terms of odds ratios expressed as $\exp (\upbeta \cdot\Delta {\text{X}})$. For example a 10,000€ increase in economic status decreases the relative risk of substantiated child maltreatment by 17%; a 0.1 increase in education level decreases the relative risk by 15%; a 5 unit increase in policing activity increases the relative risk by 17%; finally, a 10% increase in immigrant concentration increases the relative risk by 9%.

**Fig. 1:**
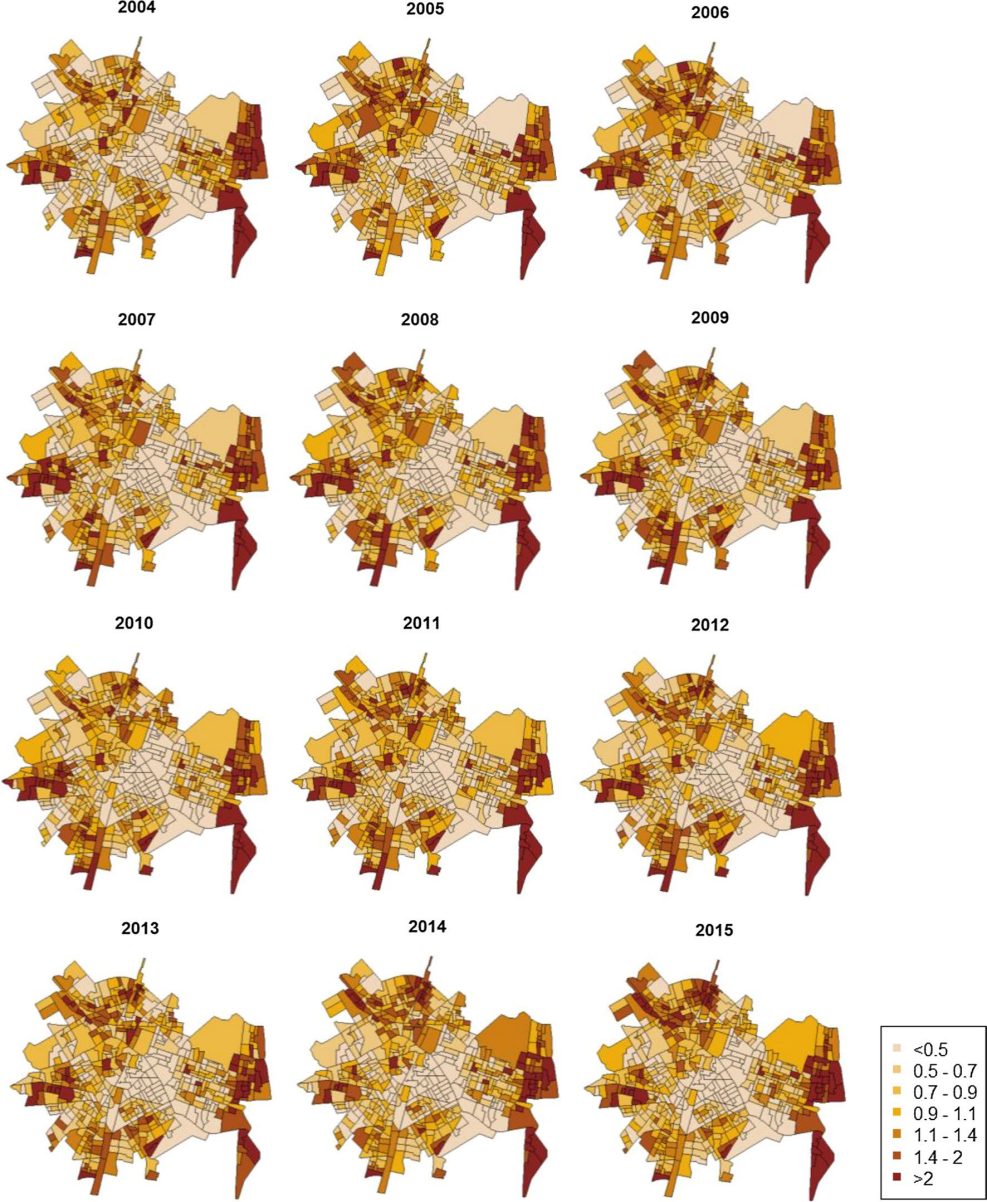
Maps of relative risks of child maltreatment by census block group in each year of study, Valencia, Spain, 2004–2015

Bayesian spatio-temporal modeling allows area-specific risks of substantiated child maltreatment to be mapped and differences analyzed over the years. Figure 1 shows the relative risk for each year of the study. These maps show areas with higher (> 1) or lower (< 1) than average risk. In some areas, the relative risk is more than twice the average, which reflects very high-risk levels of substantiated child maltreatment.

These maps also reveal common patterns over the years, showing areas with higher levels of relative risk at the periphery, especially in the eastern part of the city. The parameter $\rho$ ($\rho$ = .90) indicated a high temporal correlation between a particular year and the previous one.

**Fig. 2:**
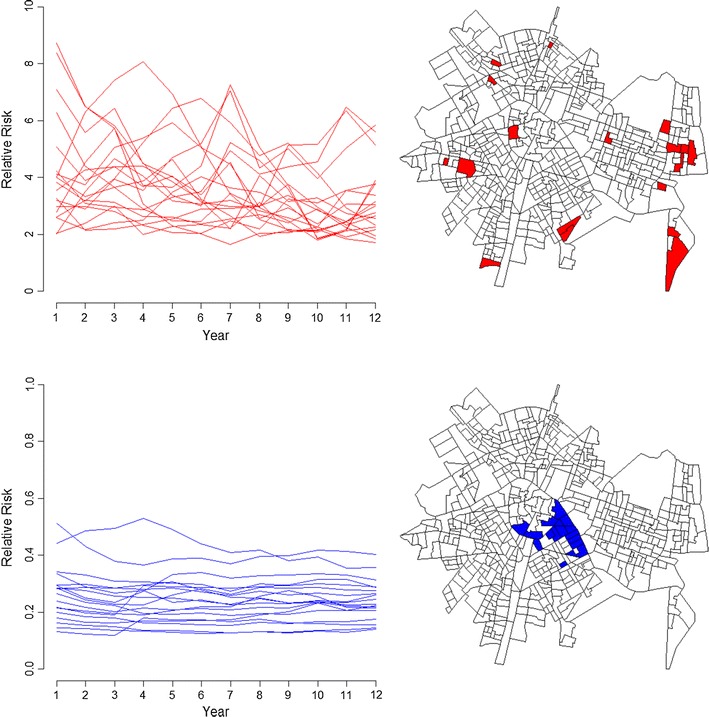
Temporal paths of relative risk in areas with stable high risk (above), and stable low risk (below). Relative risk values greater than 1 indicate higher risk than the city average. Relative risk values lower than 1 indicate lower risk than the city average

**Fig. 3:**
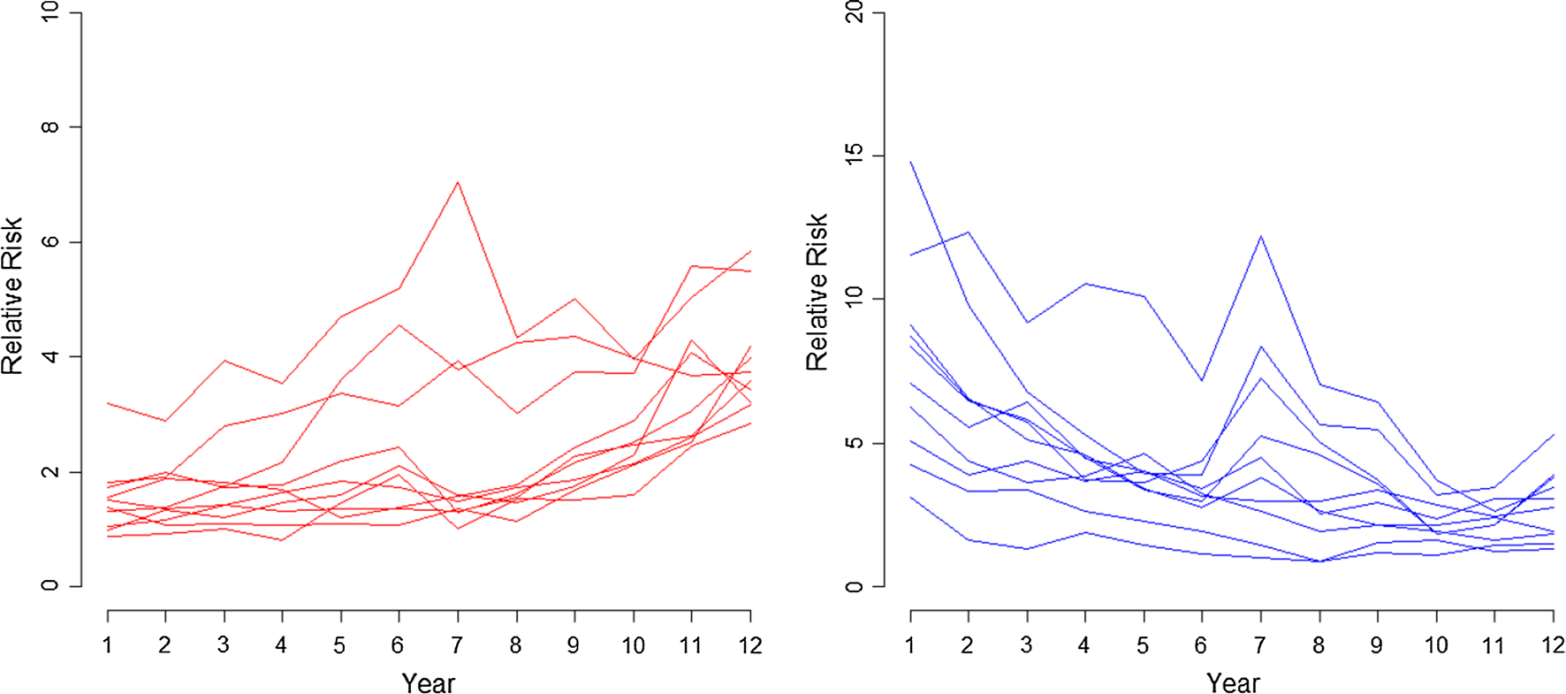
Temporal paths of relative risk in areas with increasing and decreasing child maltreatment risk, respectively

Autoregressive models can be used to represent temporal paths of relative risk in different census block groups, thus identifying areas with stable risks and areas with changes in risk over time. Figure 2 shows the most stable areas. Results showed both chronic spatial patterns of high risk of substantiated child maltreatment during the years analyzed, as well as stability over time in areas of low risk. The most stable low risks are located in the city center, while peripheral areas present more stable high risks. Figure 3 shows areas with increased or decreased substantiated child maltreatment risk over the years.

## Discussion

This study showed that the neighborhoods where the families live matter in understanding spatial and temporal variations in child maltreatment risk [8–12]. Our results showed that neighborhoods with low levels of economic and educational status, with high levels of policing activity, and high immigrant concentration had higher risk of substantiated child maltreatment. This study illustrates how the unequal distribution of neighborhood risk factors in an urban area is linked to the unequal spatial distribution of substantiated child maltreatment risk in the city. These results support the view that ‘place’ matters in relation to child maltreatment, as these ecological variations reflect important inequalities in substantiated child maltreatment risk [10–12]. Among the explanatory mechanisms that have been proposed to explain this link are: social impoverishment—that is, lack of trust and support networks in the neighborhood—, and diminished social control; social isolation from mainstream values regarding what is acceptable in parent–child relationships; and high levels of parental stress [8, 10–12, 19, 27].

Previous studies have linked these neighborhoods characteristics—that is, neighborhood socioeconomic disadvantage, disorder and crime, and immigrant concentration—indicative of social disorganization to child maltreatment rates [10, 11, 27, 29]. However, the present study is, as far as we are aware, the first to analyze this link using a high-resolution Bayesian spatio-temporal approach to study the influence of neighborhood characteristics on small-area variations in substantiated child maltreatment risk. Using this approach, we were able to analyze small-area variations in substantiated child maltreatment risk over a 12-year period, which provided the possibility to identify and track risk trends. Our results, in addition, show that ‘time’ is also a key element to analyze variations in child maltreatment risk. Studies that do not take into account the temporal dimension may misinterpret risk estimations due to data aggregation—for example increasing or decreasing risks in certain neighborhoods would not be detected. In our study, introducing a spatio-temporal autoregressive structure clearly improved the model fit, and provided more reliable area-specific risk estimates. Thus, our results showed chronic spatial patterns of high risk of substantiated child maltreatment during the years analyzed. We found areas where risks were over five-times higher than the city average, and this high risk was stable over the years. Results suggest that in certain city areas substantiated child maltreatment risk can become ‘endemic’. On the other hand, risks in other areas were between two and four times lower than the city average, and these lower risks were also stable over the years. In this regard, low-risk city areas could be considered to be providing a more protective social environment for the well-being of children, as opposed to the very high-risk ones—for example, differences in risk between some of the low risk areas and some of the high-risk ones are nearly tenfold. Finally, spatio-temporal analyses allowed us to identify areas where substantiated child maltreatment risk increased or decreased over the years analyzed.

Detecting and tracking these inequalities in substantiated child maltreatment risk is important for better-informed prevention and intervention strategies. One of the advantages of the high-resolution approach used in the present study is that it provides small-area-specific risk estimates pointing to areas of stable excess risk, as well as to areas of increasing or decreasing substantiated child maltreatment risk. Identifying and tracking these risk trends at the small-area level can provide a more useful method to inform policy and action, as compared to other low-resolution approaches—for example, counties or large census tracks. Interventions targeting areas of excess risk of child maltreatment—for example, increasing or chronic high-risk areas—can have an important preventive potential. Neighborhood-level interventions, as opposed to a person-centered approach, can reach a larger number of families, providing a more cost-effective strategy, as not only the individuals are the subject of the intervention, but also the context where these families live [10, 55, 56]. The spatio-temporal epidemiological approach used in this study not only provides a powerful method to map and track variations in risk, but it can also contribute to a surveillance system to assess the effectiveness of intervention and prevention strategies by monitoring changes in risk over time across different city areas [6, 12].

Limitations to our study include the type of data used, namely, only officially reported and substantiated cases of child maltreatment under the supervision of Child Protection Services. Child maltreatment is, however, still largely underreported and underestimated, as many cases do not come to the attention of these services, or are unsubstantiated after reporting [4, 44]. On the other hand, it is important to note that families living in high risk neighborhoods may be more visible to authorities and therefore can be more susceptible to be reported and substantiated, as these residential areas may lead to a higher surveillance by social welfare or law enforcement agencies, as compared to other residential areas [10, 11, 42, 44]. A related issue is the potential problem of neighborhood selection bias, whereby families with higher risk of being investigated by child protection services, either choose or are forced to live in these high-risk neighborhoods [10]. The question here is to which extent the higher risk of substantiated child maltreatment is the result of the influence of neighborhood-level factors or the self-selection of families with certain characteristics in certain neighborhoods. Although this line of criticism tends to give more preeminence to individual-based explanations, than to other higher-order explanations such as neighborhood mechanisms, a substantial body of research supports the link between neighborhood concentrated disadvantage and the spatial inequality in a wide variety of outcomes, including violence, crime, or health. This body of literature led Sampson to conclude that “spatially inscribed social differences, …, constitute a family of neighborhood effects that are pervasive, strong, cross-cutting, and paradoxically stable even as they are changing in manifest form” [55, p. 6].

Regarding the covariates used in our study, the measurement of policing activity could be biased by self-report. However, this measure has adequate psychometric characteristics [49, 50] and previous studies have shown that police perceptions capture their valuable experience, are correlated with police records, and can provide important information to identify high crime areas [50, 57–59]. Nevertheless, clearly, future studies would benefit from using more objective crime reports. Another limitation in our study is that a number of potentially relevant neighborhood-level measures were not available for a 12-year spatio-temporal analysis. For example, socioeconomic measures such as people living below the poverty line, rates of unemployment, or income, or other covariates tapping potentially relevant neighborhood processes—such as collective efficacy, social networks, neighborhood disorder, neighborhood social norms, or alcohol outlets—, were not available [29–31, 60–66]. The modifiable areal unit problem is also a potential limitation. We are confident, however, that the high-resolution approach in this study, using the smallest geographical administrative unit available—census block groups are walkable areas with a small number of city blocks—, was particularly adequate to capture neighborhood influences on small-area variations in risk [7].

Regarding the implications for policy and action, the neighborhood conditions linked to substantiated child maltreatment in the present study are risk factors that cluster in space and, therefore, can be targeted in more focused preventive interventions. Although potentially modifiable, some of these factors, such as poor housing, or high levels of crime, or high levels of immigrant concentration are clearly difficult to change. However, other type of focused neighborhood-level interventions can address indirectly these factors. For example, urban planning and environmental approaches such as urban redevelopment and revitalization—for instance, providing new infrastructures, greening vacant lots, improving access to services, or increasing community programs—, have been shown to improve the quality of life of residents, and reduce crime, drug use, and violence in disadvantage communities [67–70].

Finally, this study was conducted in a medium-sized European city. Although similar neighborhood effects in relation to other type of offenses have been observed in European and American cities despite their differences in culture and organization [43, 55, 71, 72], future cross-national research with a similar approach would help to strengthen and generalize our results.

## Conclusion

Our 12-year study showed that there are important spatial inequalities in substantiated child maltreatment risk across the city areas and over the years. Our study illustrates that a spatio-temporal epidemiological approach to study the geographical patterns, trends over time, and the contextual determinants of substantiated child maltreatment risk, can provide a useful method that can be of help to better inform policy and action. This methodological approach—that uses data that can be routinely collected—, can offer a more accurate description of the problem, and help to design new local prevention and intervention strategies. A spatio-temporal approach can also contribute to an improved epidemiological surveillance system to detect ecological variations in risk, and to assess the effectiveness of the initiatives to reduce this risk.
